# Phase contrast micro-CT with adjustable in-slice spatial resolution at constant magnification

**DOI:** 10.1088/1361-6560/ad4000

**Published:** 2024-05-07

**Authors:** Amir Reza Zekavat, Grammatiki Lioliou, Oriol Roche i Morgó, Charlotte Maughan Jones, Gabriel Galea, Eirini Maniou, Adam Doherty, Marco Endrizzi, Alberto Astolfo, Alessandro Olivo, Charlotte Hagen

**Affiliations:** 1 University College London, Department of Medical Physics and Biomedical Engineering, London, United Kingdom; 2 University College London, GOS Institute of Child Health, London, United Kingdom

**Keywords:** phase contrast, computed tomography, resolution

## Abstract

*Objective.* To report on a micro computed tomography (micro-CT) system capable of x-ray phase contrast imaging and of increasing spatial resolution at constant magnification. *Approach.* The micro-CT system implements the edge illumination (EI) method, which relies on two absorbing masks with periodically spaced transmitting apertures in the beam path; these split the beam into an array of beamlets and provide sensitivity to the beamlets’ directionality, i.e. refraction. In EI, spatial resolution depends on the width of the beamlets rather than on the source/detector point spread function (PSF), meaning that resolution can be increased by decreasing the mask apertures, without changing the source/detector PSF or the magnification. *Main results.* We have designed a dedicated mask featuring multiple bands with differently sized apertures and used this to demonstrate that resolution is a tuneable parameter in our system, by showing that increasingly small apertures deliver increasingly detailed images. Phase contrast images of a bar pattern-based resolution phantom and a biological sample (a mouse embryo) were obtained at multiple resolutions. *Significance.* The new micro-CT system could find application in areas where phase contrast is already known to provide superior image quality, while the added tuneable resolution functionality could enable more sophisticated analyses in these applications, e.g. by scanning samples at multiple scales.

## 1. Introduction

Micro computed tomography (micro-CT) is an x-ray based imaging method that has gained popularity in biomedical disciplines (Rawson *et al* 2020, Withers *et al* 2021). Owing to their short wavelength, x-ray photons can penetrate through bulk samples and tissue, making them suitable to specimens which are unattainable for common optical approaches. Moreover, micro-CT images are inherently 3D, visualising details in their greater volumetric context.

The technology is constantly evolving (Clark and Badea 2021), with efforts dedicated towards maximising key image quality indicators such as contrast-to-noise ratio (CNR) (Dudak *et al* 2016) and spatial resolution (Dreier *et al* 2021). At constant noise, CNR is determined by contrast, which in micro-CT is derived from differences in the attenuation of x-rays by the tissues they pass through. Consequently, strongly attenuating tissue in a weakly attenuating background generates strong contrast, but weakly attenuating tissue in a weakly attenuating background tends to generate only low contrast, to the extent that a detail may ‘vanish’ in the noise floor. To improve the contrast between weakly attenuating materials, x-ray phase contrast imaging (XPCi) has been developed (Bravin *et al* 2013, Endrizzi 2018). The term refers to a class of imaging approaches that exploit the phase shift of x-rays, which are linked to a sample’s refractive properties, alongside their attenuation. XPCi can detect minute tissue variations, making it well suited to the imaging of unstained biological soft tissue (Zdora *et al* 2017, Toepperwien *et al* 2018, Hellerhoff *et al* 2019, Katsamenis *et al* 2019). Spatial resolution in micro-CT is first and foremost determined by the x-ray source and the detector via their point spread functions (PSF). When the detector PSF dominates, resolution can be increased by positioning the sample closer to the source (Thompson *et al* 2018). This increases magnification, that is, makes features appear larger in the detector plane, but it also demagnifies the available field of view (FOV). Vice versa, when the source PSF dominates, resolution can be increased by positioning the sample close to the detector (Toepperwien *et al* 2017). Magnification is now close to 1, but the FOV is again limited, this time by a relatively small detector pixel matrix. Therefore, while high-resolution scans can be achieved, these are typically confined to increasingly small samples. To image larger samples at high resolution, so-called region of interest tomography may be applied to local sample areas (Luo *et al* 2018). Several region-of-interest tomograms can then be stitched together to form large, high-resolution images (Kyrieleis *et al* 2009), but stitching artefacts can occur (Borisova *et al* 2021). Developments in detector technology allow to physically enlarge the FOV by combining several pixel matrices (butting); however, this is may not always be practical or it may be associated with a high cost.

We here report on a micro-CT technology which facilitates XPCi (i.e. improves soft tissue contrast) while also allowing to increase spatial resolution without encountering the challenges described above. The approach is based on the edge illumination (EI) technique (Olivo 2021), which is one of few XPCi approaches that can be implemented effectively with relatively weakly coherent x-ray sources and in compact laboratory setups (Olivo and Speller 2007). The technique’s working principle will be reviewed below but, briefly, it involves structuring the x-ray beam into array of narrow ‘beamlets’ by means of masks with transmitting apertures through which x-ray refraction can be detected, enhancing contrast. Spatial resolution in EI depends on the mask aperture width rather than the source/detector PSF, a feature referred to as ‘aperture-driven resolution’, provided there is no, or only minimal, overlap between adjacent beamlets (Diemoz *et al* 2014, Hagen *et al* 2018). This has the advantage that resolution can be increased by decreasing the width of the apertures without operating at a high magnification or using small pixels. That is, aperture-driven resolution is not intrinsically linked toa decrease in the FOV, as it does not rely on enlarging features prior to detection or on resolving minute spatial differences in the incident wavefront, but rather it restricts the area from which information can arise, providing increasingly localised signals. The concept has already been exploited for planar x-ray microscopy (Endrizzi *et al* 2014, Esposito *et al* 2022), but a micro-CT system capable of XPCi and featuring aperture-driven, and thus easily adjustable, spatial resolution had not yet been realised. Here, we report on the design and performance of such a system. The key innovation is the usage of a dedicated mask, featuring multiple discrete bands with increasingly small apertures, to create the beamlet array.

The new system could find application in areas where XPCi is already known to provide superior image quality. One such area is intra-operative specimen imaging, which involves micro-CT scanning of excised tumour-bearing tissue to establish the tumour margin status, ideally directly within the operating theatre. This application benefits from XPCi (Massimi *et al* 2021), and the added aperture-driven resolution functionality could enable even more sophisticated analyses, e.g. by scanning samples at multiple scales. A second example, which is illustrated in section 3.2, is to use the new system to image mouse embryos, which are commonly used for phenotyping, i.e. the observation of changes in certain characteristics (e.g. morphology) in response to genetic or environmental alterations. Conventional micro-CT can image mouse embryos but typically requires iodine staining to obtain sufficient contrast (Horner *et al* 2021, Handschuh and Gloesmann 2022). Since iodine staining is incompatible with histological staining, different embryos need to be collected for histological work-up. A stain-free approach, as offered by XPCi, would allow to use the same embryo, thus removing inter-subject variability and ultimately reducing the number of experimental animals needed. The new aperture-driven resolution functionality provides the necessary resolving power for this application.

Besides demonstrating the functionality of the system, a second focus of this article is on its effective operation. Aperture-driven resolution comes at the cost of a reduced sample exposure, as a significant fraction of photons is absorbed by the opaque mask septa. Moreover, the beam structuring causes parts of the sample to remain ‘unseen’, as the beamlets pass through the sample only in discrete locations. To maintain baseline photon statistics and allow the entire sample to contribute to the image, it is necessary to acquire multiple frames, each one with the sample shifted by a sub mask period distance and stitching the frames together in an interleaved fashion (a process referred to as ‘dithering’) (Hagen *et al* 2018). While dithering can be realised relatively easily for a planar image, in a CT scan, where hundreds or thousands of projections must be acquired, it can lead to long scan times. Therefore, scan times in mask-based systems tend to be longer than in conventional micro-CT systems where no masks are used. While relative scan times between both types of systems cannot be generalized as this would depend on various factors including the x-ray source and the detector, in simplified terms the extra scan time required in a mask-based system is given by the number of dithering steps, which, in turn, can be calculated from the ratio between the mask period and aperture width. Since our instrument is not only meant to enable adjustable resolution but also be practicable and usable in a variety of contexts, we explore options for implementing faster scans, including with a reduced total exposure, based on recently developed fly scan acquisition schemes (Hagen *et al* 2020) and machine learning based denoising (Hendriksen *et al* 2020).

## 2. Methods

### 2.1. Working principle of edge illumination XPCi

**Figure 1.**
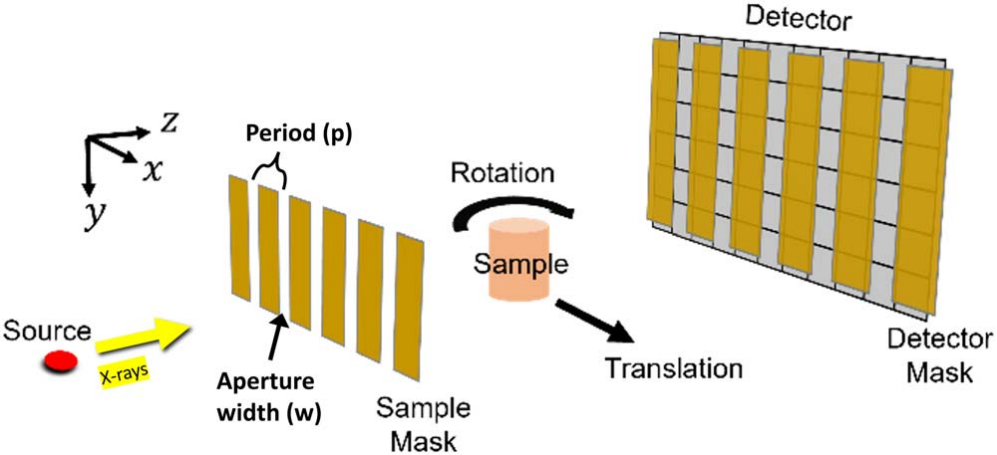
Illustration of an EI micro-CT setup. The masks shown are an illustrative example; a core element of the work reported here was to design a new sample mask allowing to adjust resolution in a flexible manner, with key design parameters being the width (*w*) of the mask apertures and the period (*p*). To acquire fully sampled datasets the sample must be translated along the lateral (*x*) direction and frames acquired at multiple sub-period positions to compensate for the undersampling in a single frame due to the strongly structured x-ray beam.

Edge illumination is an XPCi technique which makes use of two absorbing masks with periodically spaced slit-shaped transmitting apertures in the beam path (figure 1). The first mask, which is known as ‘sample mask’ and placed immediately upstream of the sample stage, structures the x-ray beam into an array of narrow ‘beamlets’. The second mask, known as ‘detector mask’, is positioned in front of the detector such that it covers the junctions between adjacent pixel columns while leaving their central areas exposed. The masks are often designed such that they provide one beamlet for each detector pixel column (i.e. the mask periods match the pixel pitch when adjusted for magnification), but they can also be ‘line-skipping’, meaning that there is one beamlet for every other pixel column, while the pixel columns in between are blocked. A line-skipping design is favourable when there is extensive crosstalk between pixels, which has a detrimental effect on the acquired images (blurring, signal diffusion) but is reduced when every other pixel column is discarded (Ignatyev *et* *al* 2011). The sample and detector masks are placed at an offset to one another, commonly such that about half of each beamlet falls into the exposed pixel area. The method derives its name from this configuration, as effectively the beamlets illuminate a series of edges created by the detector mask. When a sample is introduced, refraction causes the beamlets to slightly change their direction (in addition to being attenuated), which, in turn, makes them shift either towards, or away from, the exposed pixel areas. Consequently, more, or fewer, photons are detected relative to the reference illumination, which gives rise to contrast.

The image formation in EI can be described as follows:

\begin{eqnarray*}I(x,y)={I}_{0}\cdot \exp \left(-2k\int \beta (x,y,z;k){\mathrm{d}}z\right)\cdot C\left({x}_{m}-{z}_{{od}}\displaystyle \frac{\partial }{\partial x}\int \delta (x,y,z;k){\mathrm{d}}z\right),\end{eqnarray*}

where *I*(*x*, *y*) denotes the intensity recorded on the detector, *I*_0_ is the intensity per beamlet upstream of the sample, *k* is the wave number, *z*_*od*_ is the distance between the sample and detector, and *β*(*x*, *y*, *z*; *k*) and *δ*(*x*, *y*, *z*; *k*) are the real and imaginary parts of the complex refractive index (as per *n*(*z*, *y*, *z*; *k*) = 1 − *δ*(*x*, *y*, *z*; *k*) + *i* *β*(*x*, *y*, *z*; *k*) with *i* the imaginary unit) which describe the attenuating and refractive properties of the sample, respectively. The function *C*(.), often referred to as illumination curve, is system specific and describes the intensity modulation that results from the beamlet shifts caused by refraction. The curve is measured ahead of a scan without a sample in place, by stepping the sample mask in small steps along the *x*-axis, recording the detected intensity, and then plotting this against the sample mask position (often, the illumination curve is approximately Gaussian shaped). The *x*-axis position *x*_*m*_ denotes the position of the the sample used for imaging; as described above, this is typically chosen such that approximately half of each beamlet falls into an exposed pixel area, corresponding to a slope of the illumination curve.

Due to the beam structuring, the intensity described by equation (1) is measured in discrete points corresponding to the locations in which the beamlets traverse the sample, which leads to ‘gaps’ along the *x*-direction as no intensity measurements are available for sample areas covered by the absorbing mask septa. To obtain measurements for these areas, so-called dithering needs to be implemented, by which the sample is translated along *x* in sub-mask period steps, intensity measurements are acquired at each step and interleaved to create a complete dataset. To implement a tomographic scan, the sample is rotated along the *y*-axis, and dithering is applied at each rotation angle.

To reconstruct tomographic images, the acquired intensity data have to be converted into a line integral format first. This usually requires phase retrieval, which describes the process of isolating attenuation and refraction signals from the measured data, as these possess line integral relationships with *β*(*x*, *y*, *z*; *k*) and *δ*(*x*, *y*, *z*; *k*), respectively. Since the isolation of attenuation and refraction signals requires two intensity measurements at different sample mask positions (Olivo 2021) which is impractical, we have applied an approximation-based ‘single-image’ phase retrieval (Diemoz *et al* 2017). In analogy to the widely used Paganin method in propagation based XPCi (Paganin *et al* 2002), the approach is derived under the assumption of a constant relationship between *β*(*x*, *y*, *z*; *k*) and *δ*(*x*, *y*, *z*; *k*) across the sample, i.e.:

\begin{eqnarray*}\begin{array}{l}\gamma := \displaystyle \frac{\delta (x,y,z;k)}{2k\beta (x,y,z;k)}=\mathrm{const}.\quad \mathrm{for}\,\mathrm{all}\ (x,y,z).\end{array}\end{eqnarray*}

Under this assumption, and if the sample mask position *x*_*m*_ corresponds to a linear part of the illumination curve *C*(.), a signal that is compatible with tomographic reconstruction can be retrieved by applying a dedicated low-pass filter to the intensity data:

\begin{eqnarray*}\int \beta (x,y,z;k){\mathrm{d}}z=-\displaystyle \frac{1}{2k}\mathrm{log}\left({{ \mathcal F }}^{-1}\left(\displaystyle \frac{{ \mathcal F }\left(I(x,y)\right)}{1-i2\pi \left({z}_{{od}}\tfrac{C^{\prime} ({x}_{m})}{C({x}_{m})}\right)\gamma }\right)\right).\end{eqnarray*}

where ${ \mathcal F }$ and ${{ \mathcal F }}^{-1}$ denote the Fourier transform and its inverse, respectively. The retrieved signal can be thought of as a merged signal that contains contributions from attenuation and refraction, thus providing a greater CNR than achievable from attenuation alone. Even though the assumption of a constant *γ* (equation (2)) appears strong and essentially demands that a sample is made from a single material, it is reasonably well fulfilled for samples composed of similar materials, which is often the case for biological soft tissue samples.

### 2.2. Experimental setup

Our micro-CT system features a MicroMax 007 HF x-ray source with a rotating molybdenum target (Rigaku, Japan), operating at 40 kVp and 30 mA. The emitted beam is polychromatic, with a mean energy of approximately 18 keV. The source focal spot measures approximately 70 *μ*m horizontally and approximately 100 *μ*m vertically (full width at half maximum). The system further comprises of a C9732DK CMOS flat panel sensor coupled to a 150 *μ*m thick CsI scintillator (Hamamatsu, Japan), featuring 50 *μ*m square pixels. The distance between the source and detector measures approximately 86 cm. The system features motorised stages at approximately 69 cm and 84 cm from the source; these accommodate the sample mask and detector mask, respectively. The sample is placed approximately 3 cm downstream of the sample mask, and supported by linear and rotational motorised stages to facilitate CT scans during which the sample is both rotated and shifted (along the lateral direction, *x*). Two goniometers, placed underneath the rotation stage, allow the precise alignment of the rotation axis with the detector pixel columns. The magnification between the sample and detector planes is approximately 1.2, and remained unchanged for all scans reported in this article. The detector mask, which was fabricated (Microworks, Germany) by electroplating gold (150 *μ*m thickness) onto a 500 *μ*m thick graphite substrate, had 17 *μ*m wide apertures and a period of 98 *μ*m, covering two pixel columns when projected to the detector; it was therefore line-skipping. A dedicated multi-band sample mask was designed to enable adjustable aperture-driven resolution, which is described in the next section. The key experimental parameters of the setup are summarised in table 1.

**Table 1.** Key experimental parameters relating to the setup of the presented micro-CT system.

**SETUP PARAMETERS**	
X-ray source	MicroMax 007 HF (Rigaku, Japan)
Energy spectrum	Polychromatic (Mo, 40 kVp, 30 mA); mean energy approximately 18 keV
Detector	C9732DK CMOS flat panel (Hamamatsu, Japan)
Pixel matrix	2400 × 2400 pixels
Pixel size	50 *μ*m × 50 *μ*m
Source to detector distance	86 cm
Source to sample mask distance	69 cm
Sample mask to sample distance	3 cm
Source to detector mask distance	84 cm
Geometric magnification factor	1.2
Sample mask apertures	Different sizes and shapes available, see section 2.3 on multi-band sample mask
Sample mask period	79 *μ*m (line-skipping)
Detector mask apertures	17 *μ*m (slits)
Detector mask period	98 *μ*m (line-skipping)
Effective field of view (FOV)	4.5 cm×1 cm (extendable if required)

### 2.3. Multi-band sample mask

**Figure 2.**
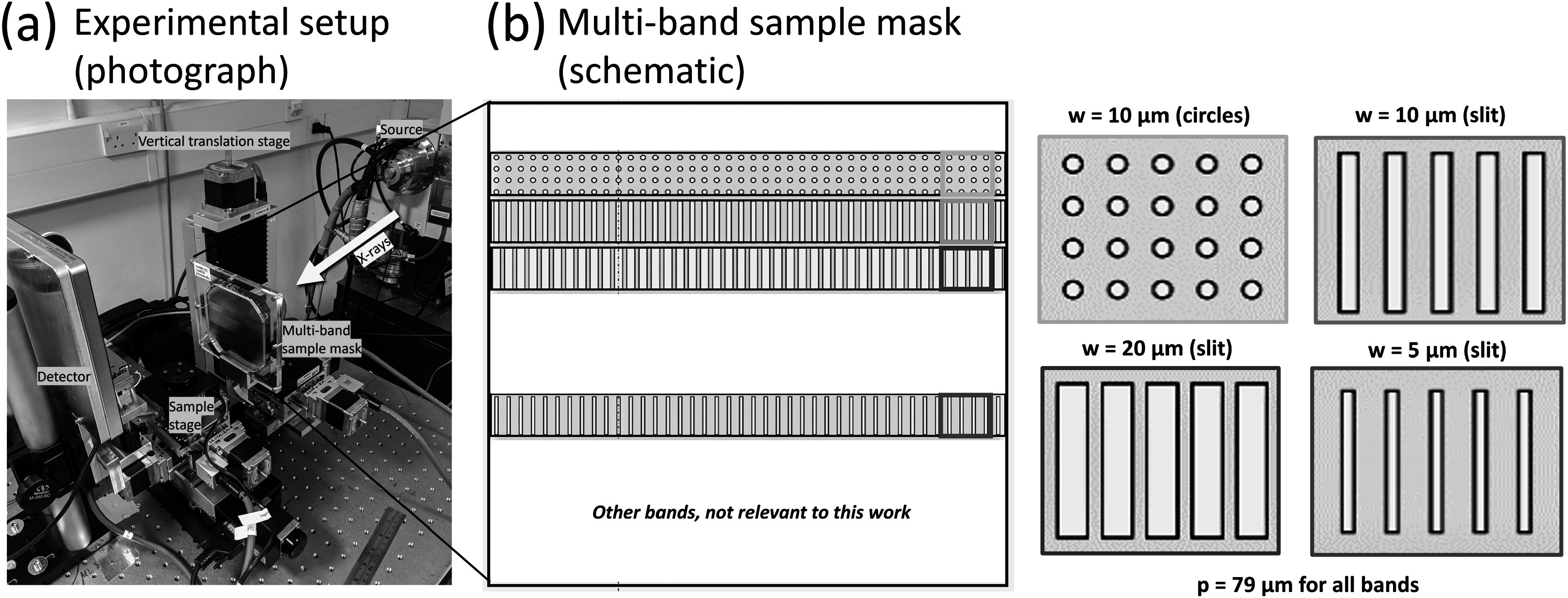
(a) Photograph showing the EI micro-CT setup with the multi-band sample mask. The photograph further shows the x-ray source, the detector, and the sample stage. A detector mask is present but cannot be seen. The x-rays’ propagation direction is indicated. The multi-band mask is situated on a vertical translation stage allowing to position a specific band for imaging. (b) Schematic of the multi-band mask, highlighting the bands that were used in this work.

To achieve adjustable aperture-driven resolution with our setup, we designed a sample mask comprising of multiple bands with progressively narrower apertures (figure 2). The mask was fabricated according to our design by electroplating gold (100–145 *μ*m thickness) onto a 200 *μ*m thick silicon substrate (Microworks, Germany). The bands with relevance to this manuscript featured vertical slits with a width (*w*) of 20, 10, and 5 *μ*m. A further band featuring circular apertures 10 *μ*m in diameter was used, which will be further elaborated upon in section 3.3. In all bands, the mask period (*p*) was 79 *μ*m, corresponding to two pixel pitches when projected onto the detector (line-skipping). Each band is 10 cm wide and 1 cm high. The horizontal FOV is defined by the diameter of the x-ray cone (approximately 4.5 cm) and the vertical FOV by the band height. Since magnification remained constant for increasing resolution, the FOV also remained constant. The band height of 1 cm was chosen so as to accommodate multiple bands on a single wafer; however, it would be technically feasible to extend each band to a larger height (e.g. matching the x-ray cone diameter) should a larger vertical FOV be required. A vertical translation stage was employed to move a specific aperture band in place for imaging.

### 2.4. Scanning schemes

Most scans were performed using the dithering scheme described above, by which the sample is shifted along the lateral direction (*x*) in several sub-period steps for every fixed angular position; that is, several frames are collected for each rotation angle. The number of frames per angle (dithering steps, *N*_dith_) needed, which also defines the sample shift per frame, depends on the ratio between aperture width and period, as this determines the extent of the under-sampling that needs to be recovered; that is: *N*_dith_ = *p*/*w*. Therefore, more frames are needed for smaller values of *w* (i.e. narrower apertures), which prolongs scan times. This is exacerbated by the fact that the higher spatial frequencies transferred by narrower apertures also require a finer angular sampling. Furthermore, dithering necessitates employing a step-and-shoot scanning sequence, which incurs scan time overheads. For these reasons, dithered scans tend to be long, especially when using a sample mask band with relatively narrow apertures.

Since our overarching aim is to develop an imaging technique that is not only capable of acquiring high-quality images, but that is also suitable for high-throughput use, there is a strong need for reducing scan time. Faster scans have the added advantage that the demands on sample stability are relaxed; this is important for soft biological samples in particular as these have a tendency to shrink, dry, or deform during very long scans. To achieve a reduction in scan time, we have trialled more time-efficient scanning methods for a subset of aperture bands and samples. These implement cycloidal sampling (Hagen *et al* 2020), by which the translation and rotation of the sample are carried out simultaneously, that is, they are combined into a joint ‘roto-translation’ movement. In a dithered scan, the sample must remain in a fixed angular position while it is stepped along the lateral direction but in a cycloidal scan this is not required. Therefore, a cycloidal scan can be implemented as a fly scan (i.e. the sample can be rotated and translated continuously). The fly scan compatibility effectively eliminates the scan time overheads and, consequently, allows for faster scans.

**Figure 3.**
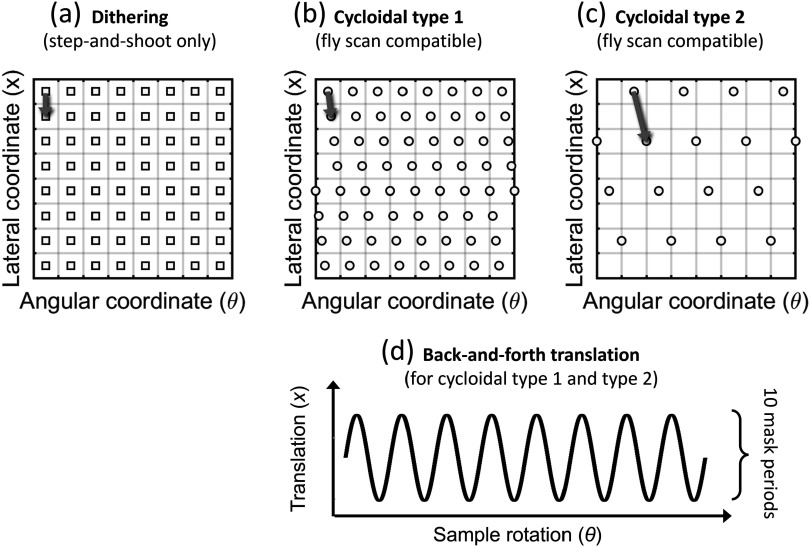
Sampling grids corresponding to different sampling schemes: (a) dithering, (b) cycloidal type 1, (c) cycloidal type 2), shown schematically for a small part of the projection-angle space (i.e. the sinogram domain). (d) Scan trajectory for a back-and-forth implementation of cycloidal sampling, by which the sample is translated by 10 mask periods along one direction, then translated by 10 mask periods in the other direction and so on, while continuously being rotated.

First, we implemented fully sampled cycloidal scans (referred to as ‘cycloidal type 1’ in the following), in which the total number of frames acquired is the same as in a dithered scan. That is, instead of acquiring data at *N* angles with *p*/*w* frames per angle (acquired at the different dithering steps), we acquired data at *N* × (*p*/*w*) angles without any dithering. Simultaneously with each angular increment, the sample was shifted by a lateral distance that matched the dithering step. Although individual projections are under-sampled in this case, collectively the set of projections (that is, the sinogram) constitutes a fully sampled dataset. This observation is illustrated in figure 3, where it can be seen that the sampling density is the same for a dithered (a) and a cycloidal type 1 scan (b), the only difference being that the sampling grid is slightly tilted in the latter case. Owing to its fly scan compatibility, the cycloidal type 1 approach reduces scan time by eliminating overheads from the acquisition, while the actual exposure to the sample remains the same as in the dithered case. Second, we implemented a variation of the cycloidal scheme that we call ‘cycloidal type 2’. This approach utilizes the same roto-transitional technique but is aimed at facilitating even faster scans by not only eliminating scan time overheads but also acquiring fewer frames, i.e. reducing the number of sampling points in the angle-projection space (c). It should be noted that this is not merely a sub-sampling approach, since the continuous ‘roto-translation’ of the sample acts as a low-pass filter, which has an anti-aliasing effect. Therefore, the cycloidal type 2 approach is an effective way of reducing resolution without incurring severe aliasing artefacts, applicable in situations where a scan time reduction is the priority. From a practical point of view, cycloidal type 2 scans can be implemented by increasing the speed of the sample rotation and translation relative to the detector’s frame rate. The data points missing in angle-projection space can be recovered by means of bivariate interpolation.

It should be noted that there are different ways of implementing cycloidal scans. The conceptually simplest one is to perform a unidirectional translation of the sample concurrently with its rotation, however this reduces the effective horizontal FOV, as the sample translation from one side to the other has to be accommodated in addition to the size of the sample itself. Since our goal is to maintain a relatively large FOV, cycloidal scans were instead implemented with a back-and-forth translation of the sample (Roche i Morgó 2021), resulting in an oscillating trajectory with a total horizontal span of 10 mask periods (figure 3, bottom). To track the horizontal sample position during a scan, a knife edge tracking method was employed (Roche i Morgó 2022). In brief, a razor blade is mounted onto the sample translation stage but not on the rotation stage, allowing to extract sub-period positional information by analysing shifts in the blade profile.

### 2.5. Acquisition parameters and reconstruction

All scans were performed in the EI configuration, that is, the sample mask was placed at a lateral offset to the detector mask such that approximately half of each beamlet fell into the exposed pixel areas. This corresponds to a reference illumination of around 50%. CT data were acquired according to the scanning schemes described above, using the acquisition parameters listed in table 2. For the dithered scans, flat fields were acquired every 100 angles to ensure that any drifts in the illumination could be corrected for. For the cycloidal scan, flat fields were acquired at the end of a scan. Further, for the dithered scans, a ‘random jitter’ was applied. During this procedure, the sample is placed in a pseudo randomly allocated lateral position relative to the detector and masks for each angle which helps with preventing ring artefacts from forming.

**Table 2.** Acquisition parameters applied to collect the data leading to the images shown in figures 4, 5 and 7. The magnification between the sample mask and the sample (*m*_*s*_) was 1.06. The overall scan times corresponding to these acquisitions are stated in the rightmost column.

**RESOLUTION PHANTOM**						
Apertures	Acquisition type	Angles/360°	Frames/angle	Sample shift/frame	Exposure/frame	Total scan time
20 *μ*m slits	Dith. (step-and-shoot)	1000	4	20 *μ*m × *m*_*s*_	1.2 s	3 h 59 min
10 *μ*m slits	Dith. (step-and-shoot)	1800	8	10 *μ*m × *m*_*s*_	1.2 s	13 h 51 min
5 *μ*m slits	Dith. (step-and-shoot)	2600	16	5 *μ*m ×* m*_*s*_	2 s	41 h 26 min

**MOUSE EMBRYO**						

Apertures	Acquisition type	Angles/360°	Frames/angle	Sample shift/frame	Exposure/frame	Total scan time

20 *μ*m slits	Dith. (step-and-shoot)	600	4	20 *μ*m × *m*_*s*_	1.2 s	2 h 29 min
10 *μ*m slits	Dith. (step-and-shoot)	1200	8	10 *μ*m ×* m*_*s*_	1.2 s	9 h 16 min
10 *μ*m circ.	Dith. (step-and-shoot)	1200	8	10 *μ*m ×* m*_*s*_	1.2 s	9 h 16 min
5 *μ*m slits	Dith. (step-and-shoot)	2400	16	5 *μ*m ×* m*_*s*_	2 s	38 h 28 min
20 *μ*m slits	Cyc. type 1 (fly scan)	2400	1	20 *μ*m ×* m*_*s*_	1.2 s	48 min
10 *μ*m slits	Cyc. type 1 (fly scan)	9600	1	10 *μ*m × *m*_*s*_	1.2 s	3 h 12 min
20 *μ*m slits	Cyc. type 2 (fly scan)	600	1	28 *μ*m × *m*_*s*_	1.2 s	12 min
10 *μ*m slits	Cyc. type 2 (fly scan)	1200	1	20 *μ*m ×* m*_*s*_	1.2 s	24 min

Data processing involved flat field and dark field corrections as the first step in all cases. For the dithered scans, the frames acquired at the individual stepping positions were combined into fully sampled projections for each rotation angle. Subsequently, the ‘random jitter’ was reversed to obtain geometrically correct sinograms. For the cycloidal scans, the acquired frames were treated as data points in the projection-angle space, which were interpolated onto a rectangular grid by means of bicubic splines interpolation (Roche i Morgó 2022). From this point onward, all datasets were processed in the same way. The next steps were ‘single-image’ phase retrieval (Diemoz *et al* 2017) and finally CT reconstruction using filtered back projection (FBP) with a Ram-Lak filter.

## 3. Results and discussion

### 3.1. Demonstration of adjustable, aperture-driven in-slice spatial resolution

The micro-CT system’s capability for adjusting spatial resolution via the sample mask apertures was demonstrated by scanning a commercial resolution phantom (QRM, Germany) using the multi-band sample mask. The phantom consists of a horizontal and a vertical silicon chip in a plastic cylinder of 10 mm diameter. The chips, each measuring 5 mm by 5 mm by 0.66 mm, contain bar and point patterns; the bar patterns varied in line width from 5 to 150 *μ*m, corresponding spatial frequencies of 100–3.33 lp mm^−1^. The horizontal and vertical chips can be used to assess the resolution within the axial plane (in-slice resolution) and the coronal or sagittal planes (out-of-slice resolution), respectively. For brief context, resolution in a CT dataset is not necessarily isotropic, and in our case, where the beam is structured by a mask with narrow slit-shaped apertures, it is highly anisotropic, as aperture-driven resolution is only available within the axial slices, while the out-of-slice resolution remains to be defined by the combined source-detector PSF. Therefore, the effect of the different aperture bands on resolution was assessed using only the horizontal chip.

**Figure 4.**
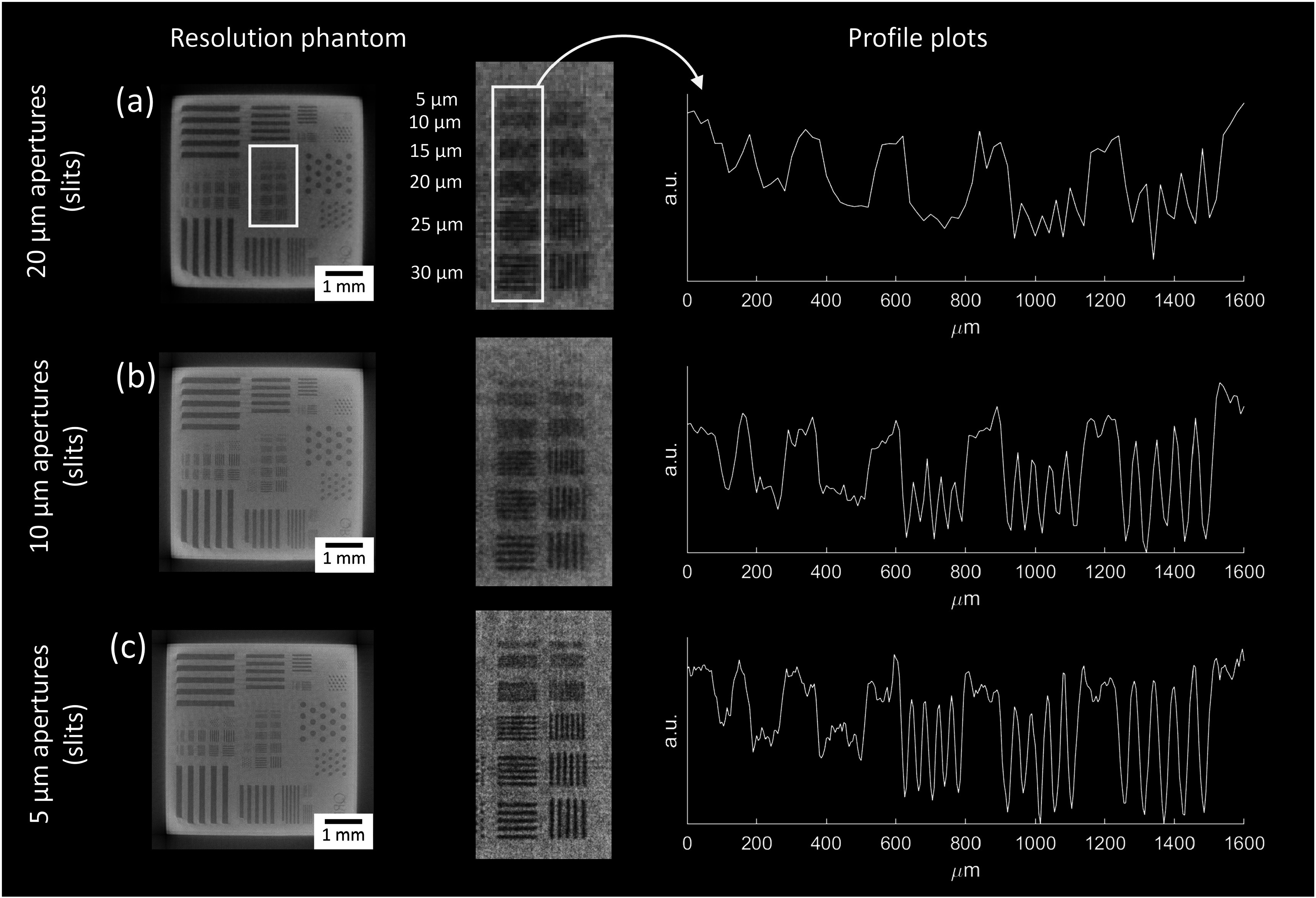
Axial slices of the QRM resolution phantom (horizontal chip) acquired with the multi-band sample mask. The zoomed areas show the central part of the chip. The rows labelled (a), (b) and (c) show images acquired with 20, 10 and 5 *μ*m apertures, respectively. All images were acquired with dithering (step-and-shoot) according to the scan parameters listed in table 2. The right hand side shows profile plots obtained by averaging horizontally over the central vertical bar patterns (the area highlighted by the yellow box).

Axial slices of the chip, acquired with the 20, 10, and 5 *μ*m aperture bands of the multi-band mask, can be seen in figures 4(a)–(c). All images were acquired with a dithered (step-and-shoot) sequence. The effect of decreasing the aperture size can be seen visually, with the images getting gradually sharper. Profile plots across the central set of vertical bar patterns, ranging from 5 to 30 *μ*m and representing spatial frequencies from 100 to 16.67 lp mm^−1^, were extracted and averaged (also shown in figure 4), from which it can be seen that the modulation improves with decreasing aperture size. While the 20 *μ*m apertures achieve some modulation for bars larger or equal to 20 *μ*m but almost no modulation for the smaller bars, the 10 *μ*m and 5 *μ*m apertures show good to excellent modulation for the larger bars and some modulation for the smaller bars. These results confirm that resolution in our approach can be controlled via the apertures in the sample mask and that resolution is therefore an adjustable parameter at constant magnification. However, it should be noted that these phantom images do not allow for measuring resolution in absolute terms. For absolute measurements, it is of crucial importance that the bar patterns (grooves) in the horizontal silicon chip are aligned with the axis of rotation and detector pixel columns, as otherwise the grooves can intersect a detector pixel at an angle, leading to an apparent loss of modulation. As our system only features goniometers underneath the sample rotation stage (to align the rotation axis to the detector pixel columns) but not above it, the phantom could only be aligned manually, an approach affected by a limited accuracy.

### 3.2. Demonstration on a sample of biomedical relevance

**Figure 5.**
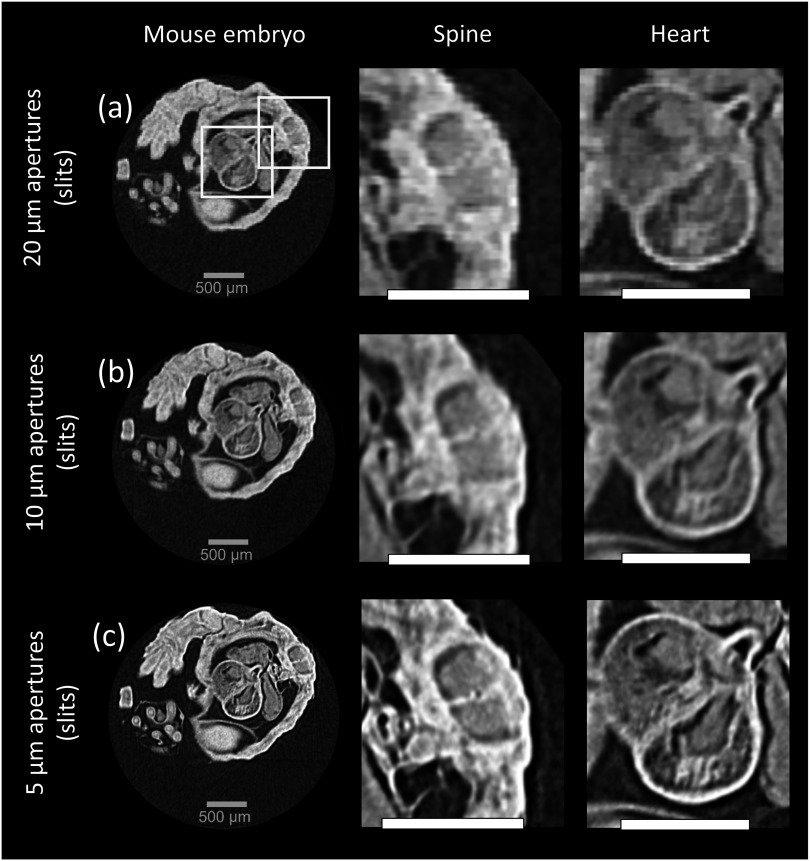
Axial slices of the mouse embryo acquired with the multi-band sample mask. The rows labelled (a), (b) and (c) show images acquired with 20, 10 and 5 *μ*m apertures, respectively. The zoomed areas show the spine and the heart. All scale bars indicate 500 *μ*m. All images were acquired with dithering (step-and-shoot) according to the scan parameters listed in table 2.

**Figure 6.**
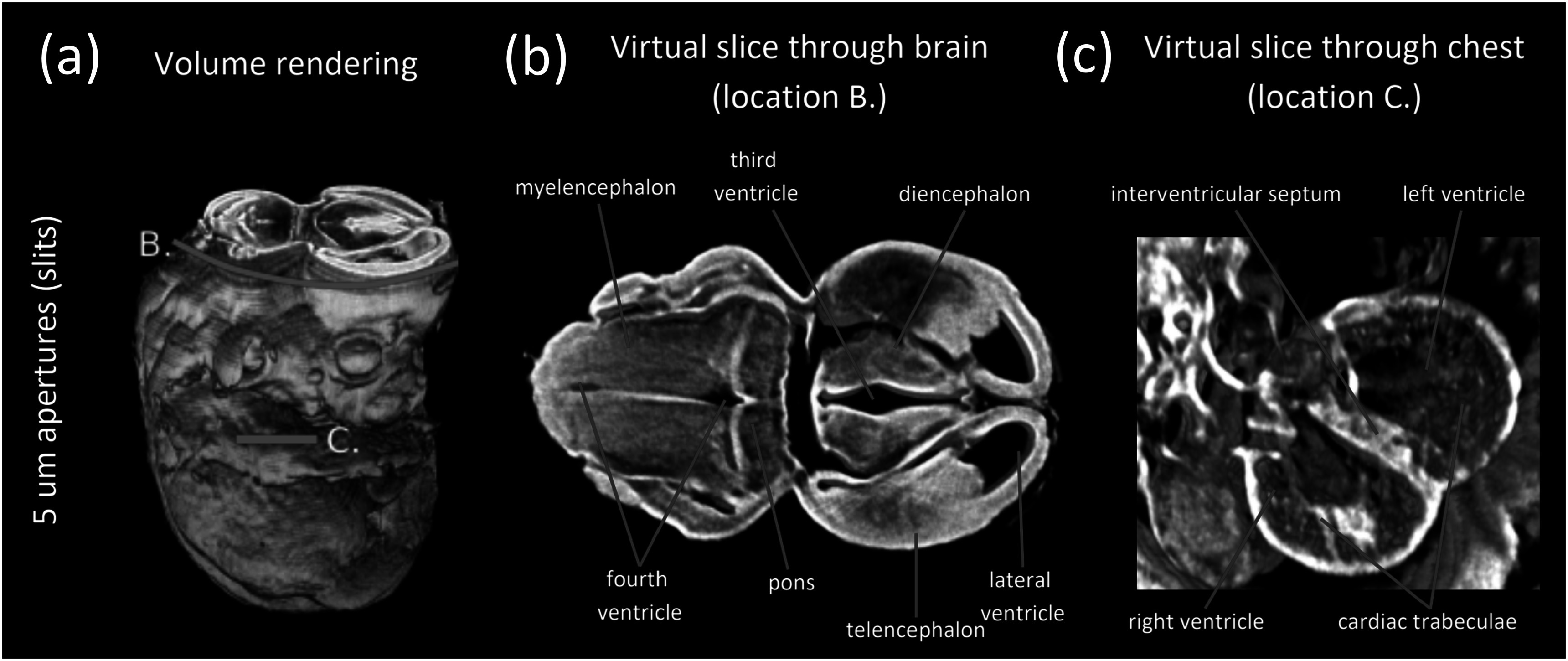
(a) Volume rendering of the mouse embryo generated from the data acquired with 5 *μ*m slit-shaped apertures (i.e. the data shown in the bottom row of figure 5). The volume was sliced at the brain and chest level (at the locations labelled B. and C.). The resulting virtual slices, which reveal intricate anatomical details, are shown in panels (b) and (c). The volume rendering and virtual slices were generated using Fiji (Schindelin *et al* 2012).

To showcase the combined performance of XPCi and aperture-driven resolution for micro-CT imaging of biological samples, we scanned a mouse embryo (C57BL6/J inbred strain wild type, 14.5 d gestation) generated as a surplus sample during research reported elsewhere (Maniou *et al* 2023). The study was performed under the regulation of the UK Animals (Scientific Procedures) Act 1986 and the National Centre for the 3Rs’ Responsibility in the Use of Animals for Medical Research (2019). Conventional micro-CT imaging of mouse embryos typically involves staining to obtain sufficient contrast, while in our case the sample was unstained. Instead, the sample was dehydrated via immersion in an escalating ethanol series ranging from 70% to 100% ethanol, following which it was left to air dry to allow full evaporation of the ethanol. This prepatation protocol, in combination with the XPCi capability of our system, led to sufficient soft tissue contrast. The sample holder was a 3D printed polylactic acid (PLA) tube of approximately 4 mm diameter, which kept the sample stable during the scans. Reconstructed slices across the mouse embryo’s thorax are presented in figure 5. All slices reveal intricate details of various internal organs, including the heart and liver, however the one acquired with the 5 *μ*m aperture band (c), benefitting from the highest spatial resolution, is clearly the most detailed. For instance, the spinal cord central canal is well resolved, while it appears blurry in the images obtained with the 10 *μ*m and 20 *μ*m apertures. The data acquired with the 5 *μ*m aperture band was used to generate a volume rendering of the mouse embryo, which is shown in figure 6. The volume was sliced at the brain and chest level, generating virtual slices of these organs and revealing intricate anatomical details.

### 3.3. Potential for scan time reduction

**Figure 7.**
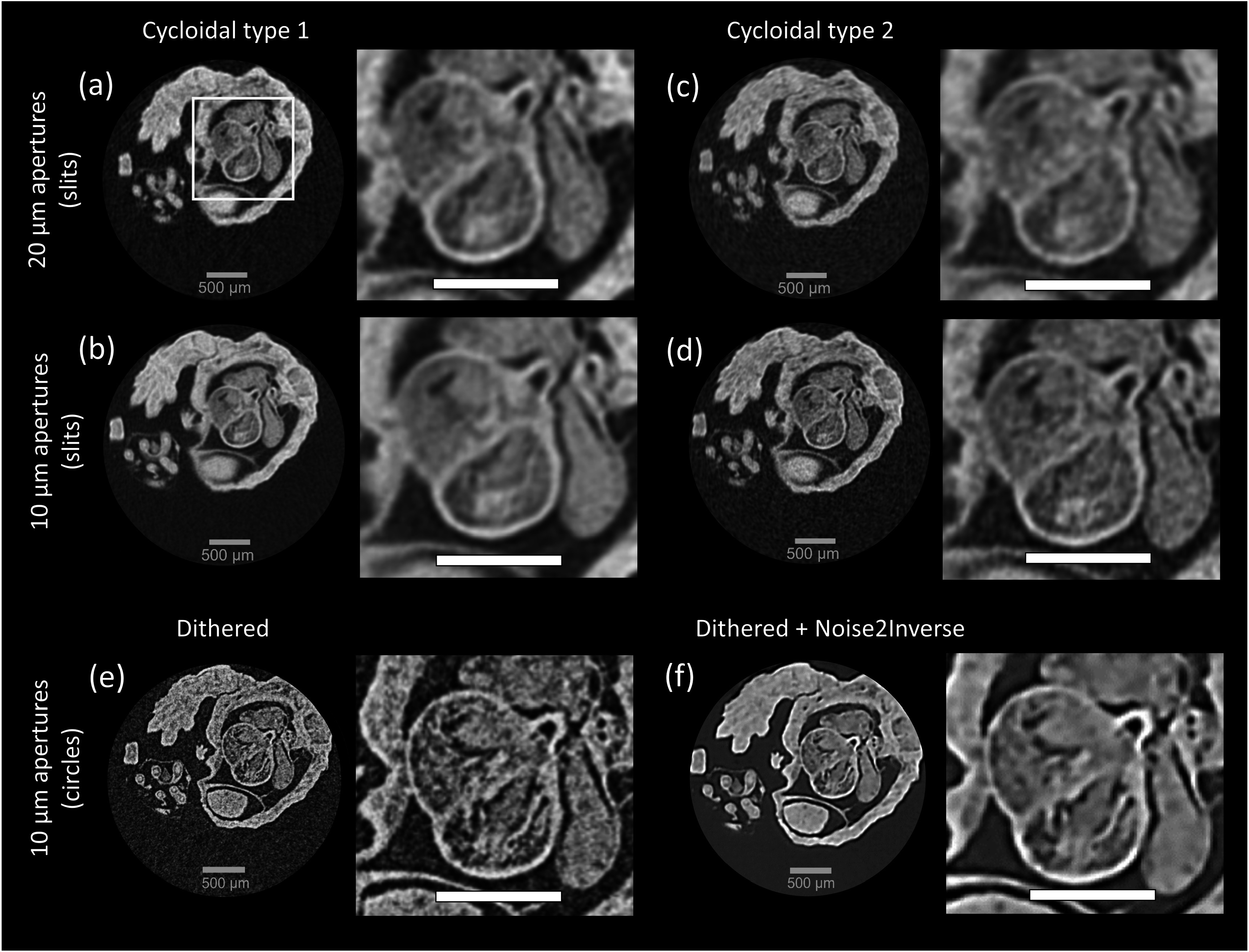
Axial slices of the mouse embryo acquired with the multi-scale sample mask. The zoomed in areas show an enlarged view of the heart. All scale bars indicate 500 *μ*m. Panels (a), (c) and (b), (d) show images acquired with 20 *μ*m and 10 *μ*m apertures, respectively. Images were acquired with cycloidal type 1 (a), (b) or cycloidal type 2 (c), (d) schemes according to the scan parameters in table 2. Panels (e) and (f) show images acquired with 10 *μ*m circular apertures and dithering (step-and-shoot) before (e) and after (f) denoising using Noise2Inverse.

As described, micro-CT scans with dithering require relatively long scan times (see table 2). To demonstrate that scan times can be reduced without significantly compromising on image quality, cycloidal scans were performed for the mouse embryo using the 20 *μ*m and 10 *μ*m apertures bands (shown in figures 7(a)–(d)). Cycloidal scans were also performed for the 5 *μ*m band but during processing it emerged that motor limitations and associated positioning issues prevented us from reconstructing high-quality images, and therefore these results are not shown. However, the 5 *μ*m aperture band is the one for which scan time reductions are needed most, hence further work will be conducted with the aim of overcoming these experimental problems. Panels (a), (b) in figure 7 show the cycloidal type 1 scans, which were acquired with the same total number of frames (i.e. the same total exposure) as the corresponding dithered scans. The resulting images are indeed very similar to their dithered counterparts, but scan times were reduced from 2 h 29 min to 48 min for the 20 *μ*m aperture band, and from to 9 h 16 min to 192 min for 10 *μ*m aperture band, respectively. Panels (c), (d) shows the results from the cycloidal type 2 scans, where, in addition to the transition from a step-and-shoot to a fly scan sequence, fewer frames were acquired. This has led to further scan time reductions, to 12 min for the 20 *μ*m aperture band and 24 min for the 10 *μ*m aperture band.

A third potential measure for reducing scan time is to shorten the exposure per frame. While taking fewer frames affects resolution as well as the overall photon statistics, a shorter exposure leads to noisier projections and, therefore, an even lower signal-to-noise ratio (SNR) in the reconstructed tomographic images. Since scans are typically already run at the minimal exposure time that yields a satisfactory SNR, this measure should be used cautiously. On the other hand, image denoising (Elad *et al* 2023), e.g. based on machine learning, provides options for restoring SNR and therefore lowering the exposure beyond conventional thresholds. Machine learning based denoising works by training a model, commonly a convolutional neural network (CNN), to differentiate between signal and noise in an image and thereby gaining the ability to remove, or reduce, the latter. Training a CNN can be supervised, whereby the model is trained on pairs of noisy input images and clean (that is, noise-free) target images, or self-supervised, where the training is performed on noisy images exclusively. The latter approach may be considered more convenient, as it does not rely on the availability of noise-free images, the acquisition of which may be impossible, or it may be dose-intensive, time-consuming, or both. Noise2Inverse (Hendriksen *et al* 2020) is a self-supervised machine learning method for denoising tomographic images. In brief, a CT dataset is split into (at least) two subsets by allocating odd numbered projections to one subset and even numbered projections to the other subset. Axial slices are reconstructed from both subsets, and the resulting slices constitute the training input and target pairs. Since the input and target images contain the same signal content but varying noise content, a CNN learns to preserve only the former when applied to a noisy input.

Our goal was to exploit Noise2Inverse to enable a substantial reduction of the exposure time. However, because some of the scans reported above were already performed close to the highest frame rate that our detector can support, we were unable to implement shorter exposure times by reducing the integration time. Instead, we used a band in our multi-band sample mask which features circular apertures (10 *μ*m diameter) to restrict the available flux and thereby mimic a low exposure acquisition. Considering the effective vertical pixel pitch in our system is 40 *μ*m, the use of circular apertures led to a >4-fold reduction in flux compared to 10 *μ*m wide slit-shaped apertures (the actual relative reduction in intensity, measured in a 15 × 15 pixel window in an area outside the sample in two dark field corrected projections acquired with the two mask bands, was approximately 9-fold). Noise2Inverse was applied by training a U-Net with an MSE loss using the 1200 dithered projections acquired; these were split into two sets of 600 projections from which pairs of axial slices were reconstructed, constituting the training input and target pairs. Training was stopped after 24 epochs and the trained network was subsequently applied to the noisy reconstructions.

The bottom row of figure 7 shows an image of the mouse embryo acquired with 10 *μ*m circular apertures before (e) and after denoising (f). The image in (e) is sharp but noisy, while the image in (f) is clearly denoised but also shows a small loss in resolution. One explanation is that by splitting the CT dataset into two subsets, the angular sampling of each subset is reduced, which can affect the resolution of the reconstructed images. To mitigate this problem, it may be beneficial to oversample the initial dataset in the angular coordinate such that, when splitting into two subsets, each subset is still adequately sampled. The oversampling would not necessarily equate to a longer overall scan time, as the exposure per frame could be halved. Further work shall be dedicated to exploring these questions in detail, as well as to comparing the performance of Noise2Inverse with traditional model-based noise reduction methods. Nonetheless, denoising appears to bear promise for further scan time reduction, especially when combined with cycloidal scanning. For example, a scan with 10 *μ*m wide slit-shaped apertures would only take a few minutes if a cycloidal scan was performed with a significantly reduced exposure per frame.

## 4. Conclusion

A micro-CT system capable of XPCi and aperture-driven resolution was presented. The key innovation lies in a dedicated multi-band sample mask, featuring increasingly narrow apertures and therefore allowing to acquire images with increasingly high resolution. XPCi facilitates high contrast, stain-free imaging of biological samples, and this was demonstrated based on images of an unstained mouse embryo. The aperture-driven resolution concept has several important additional implications. First, by introducing a mask into the setup, the source’s and detector’s PSF become significantly less relevant to the detection of high frequency signals than they would have been without the mask. Therefore, the concept enables high-resolution imaging with ‘low-resolution’ x-ray sources and detectors, i.e. those featuring relatively large focal spots and pixels. Second, resolution can be easily tailored to the sample under study by using a mask with distinctly sized transmitting apertures. In conventional systems, it is often possible to increase resolution by increasing the magnification, e.g. by bringing the sample closer the source; however, is inevitably leads to a demagnified and, therefore, compromised FOV. With aperture-driven resolution, magnification remains the same at all resolution levels, and therefore the FOV remains the same. This offers opportunities for scanning much larger samples at higher-resolution levels than with conventional approaches. Third, as a direct consequence of the flexibly adjustable resolution, multi-scale investigations can be realised by scanning a sample repeatedly with different masks, or a mask with multiple aperture bands if the sample is positioned within a different band in consecutive scans. Typically, high-resolution scans incur a higher dose than low-resolution ones and should therefore only be performed if necessary. In this context, a low-resolution scout scan could help decide whether or not a high-resolution scan is needed.

The second part of the manuscript explored options for reducing scan times. Cycloidal scans, which are known to be faster than dithering, have been implemented and demonstrated to maintain image quality. Finally, we explored the possibility of using machine learning based denoising to reduce scan times. Taken together, these measures could bring minutes-long high-resolution XPCi micro-CT scans, performed with compact laboratory setups, within the attainable range.

## Data Availability

The data cannot be made publicly available upon publication because no suitable repository exists for hosting data in this field of study. The data that support the findings of this study are available upon reasonable request from the authors.
